# Supplementary material for: Targeting fatty acid β-oxidation impairs monocyte differentiation and prolongs heart allograft survival
Source: JCI Insight. 2022 Apr 8;7(7):e151596. doi: 10.1172/jci.insight.151596 (PMC9057610; doi:10.1172/jci.insight.151596)
Supplement: Supplemental data [file jciinsight-7-151596-s196.pdf]

SUPPLEMENTAL FIGURES AND FIGURE LEGENDS

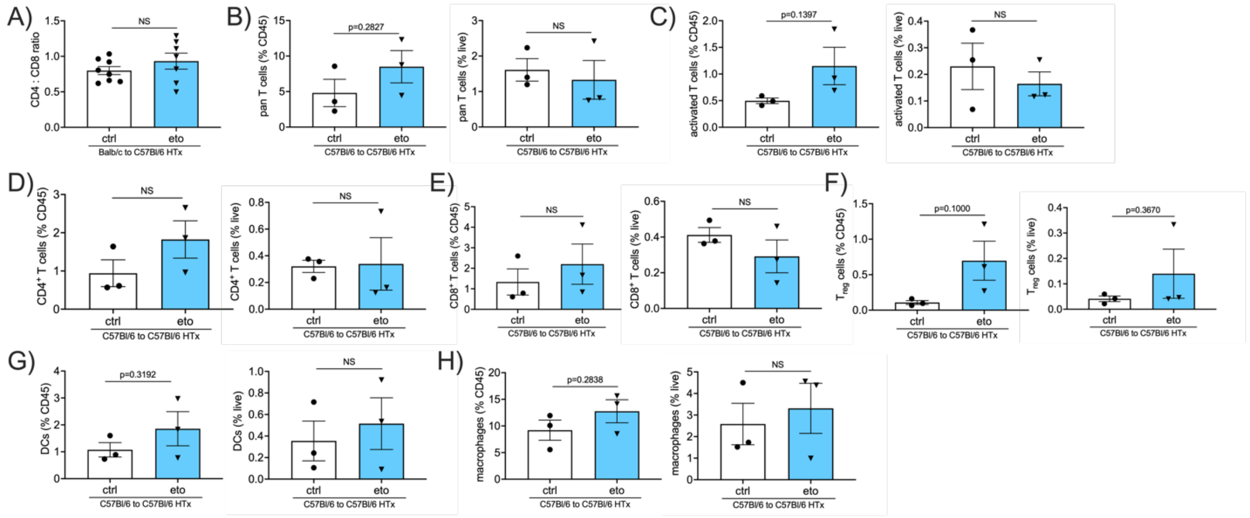

SUPPLEMENTAL FIGURE 1: CD4:CD8 T CELL RATIO IN HTX ALLOGRAFTS AND IMMUNE CELL PROFILE IN HTX SYNGRAFTS. A-H) Flow cytometric assessment in transplanted hearts from Balb → B6 heart allografts at 4 days post-transplant shown as a percentage of $CD45^+$ cells (left graph) or live cells (right graph). **A)** $CD4^+$ to $CD8^+$ T cell ratio from Balb → B6 heart allografts at 4 days post-transplant treated with vehicle (ctrl) or etomoxir (eto) (ctrl, n=8; eto, n=7). **B-H)** Flow cytometric assessment in transplanted hearts from B6 → B6 heart allografts at 4 days post-transplant shown as a percentage of $CD45^+$ cells. **B)** Pan T cells assessed by $CD3^+CD90.2^+$ cells (ctrl, n=3; eto, n=3). **C)** Activated T cells assessed by $CD69^+CD3^+CD90.2^+$ cells (ctrl, n=3; eto, n=3). **D)** CD4 T cells assessed by $CD4^+CD3^+CD90.2^+$ cells (ctrl, n=3; eto, n=3). **E)** CD8 T cells assessed by $CD8^+CD3^+CD90.2^+$ cells (ctrl, n=3; eto, n=3). **F)** T regulatory cells assessed by $Foxp3^+CD4^+CD3^+CD90.2^+$ cells (ctrl, n=3; eto, n=3). **G)** Dendritic cells (DCs) assessed by $CD11c^+MHCII^+CD64^-$ cells (ctrl, n=3; eto, n=3). **H)** Macrophages assessed by $CD11b^+CD64^+CD24^-$ cells (ctrl, n=3; eto, n=3). Data are represented as mean ± SEM. NS, not statistically significant by *t*-test.

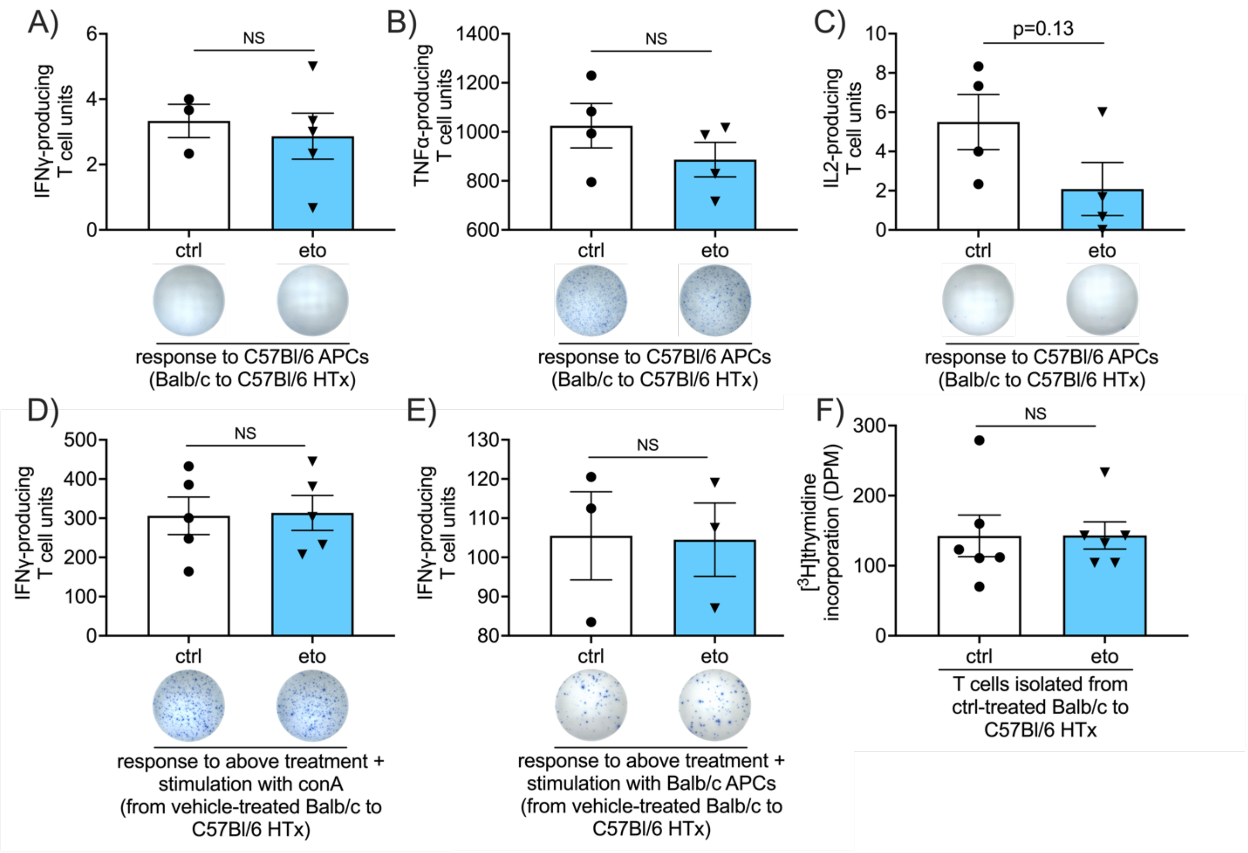

Supplemental Figure 2: T cell activation in response to B6 APCs, direct effect of FAO inhibition on T cell activation and effect of FAO inhibition on T cell proliferation. A-C) ELISPOT assay performed using splenocytes procured at 4d post-transplantation from Balb → B6 heart allografts from either vehicle (ctrl) or etomoxir (eto)-treated animals. Samples were stimulated with activated B6 APCs and assessed for the production of IFNγ (ctrl, n=6; eto, n=8) (**A**), TNFα (ctrl, n=4; eto, n=4) (**B**) or IL2 (ctrl, n=4; eto, n=4) (**C**). **D,E)** ELISPOT assay performed using splenocytes procured at 4d post-transplantation from Balb → B6 heart allografts from ctrl-treated HTx and incubated in the presence or absence of eto. **D)** IFNγ production in response to positive control stimulus (concanavalin A (conA)) (ctrl, n=3; eto, n=3). **E)** IFNγ production in response to allostimulus (Balb APCs) (ctrl, n=3; eto, n=3). **F)** Proliferation of T cells isolated from spleens from ctrl-treated Balb → B6 HTx recipients in response to treatment with ctrl or eto for 24h, as assessed by [^{3}H]thymidine incorporation (ctrl, n=6; eto, n=6). Data are represented as mean ± SEM. NS, not statistically significant by *t*-test.

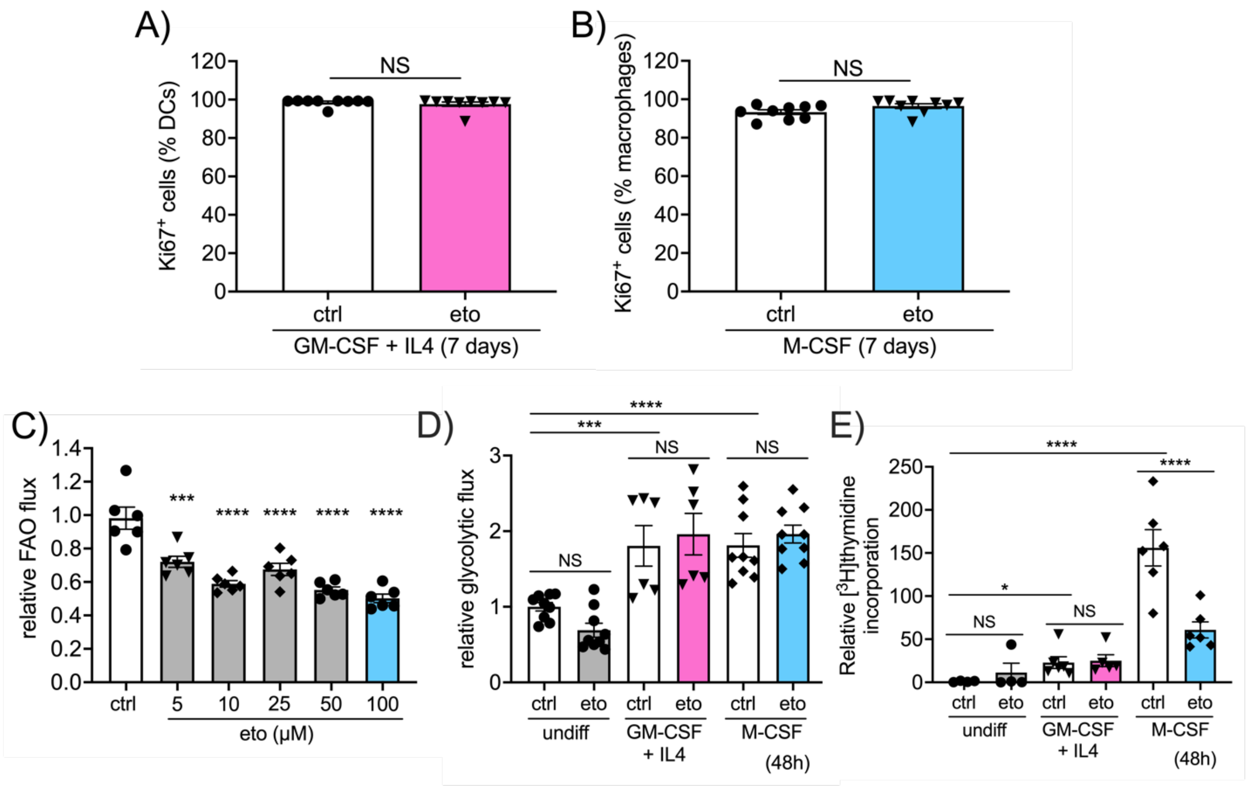

Supplemental Figure 3: Assessment of FAO inhibition on proliferation and glycolytic flux from in vitro monocyte differentiation. A-F) Data from in vitro differentiation of bone marrow-derived monocytes stimulated towards DC (GM-CSF + IL4) or macrophage (M-CSF) lineages. **A,B)** Flow cytometric assessment of cells after 7 days culture. **A)** Proliferating DCs as assessed by Ki67 as a percentage CD11c$^+$MHCII$^+$CD64$^-$ cells (ctrl, n=9; eto, n=9). **B)** Proliferating macrophages assessed by Ki67 as a percentage of CD11b$^+$CD64$^+$CD24$^-$ cells (ctrl, n=9; eto, n=9). **C)** FAO flux in monocytes treated with 5, 10, 25, 50 or 100 μM etomoxir for 24h in basal media, as assessed by 9,10-[^{3}H]palmitic acid radioisotope incorporation (n=6). **D)** Glycolytic flux in monocytes in media only, or treated for 48h with GM-CSF + IL4 or M-CSF, as assessed by [5-^{3}H]glucose radioisotopic incorporation (ctrl, n=6; eto, n=6). **E)** Proliferation of monocytes in media only, or treated for 48h with GM-CSF + IL4 or M-CSF, as assessed by [^{3}H]thymidine incorporation (ctrl, n=6; eto, n=6). Data are represented as mean of individual data points from at 3 independent experiments ± SEM. NS, not statistically significant; *p<0.05; ***p<0.001; ****p<0.0001; NS, not statistically significant, by *t*-test **(A,B)** or ANOVA and Bonferroni post-hoc test **(C-E)**.

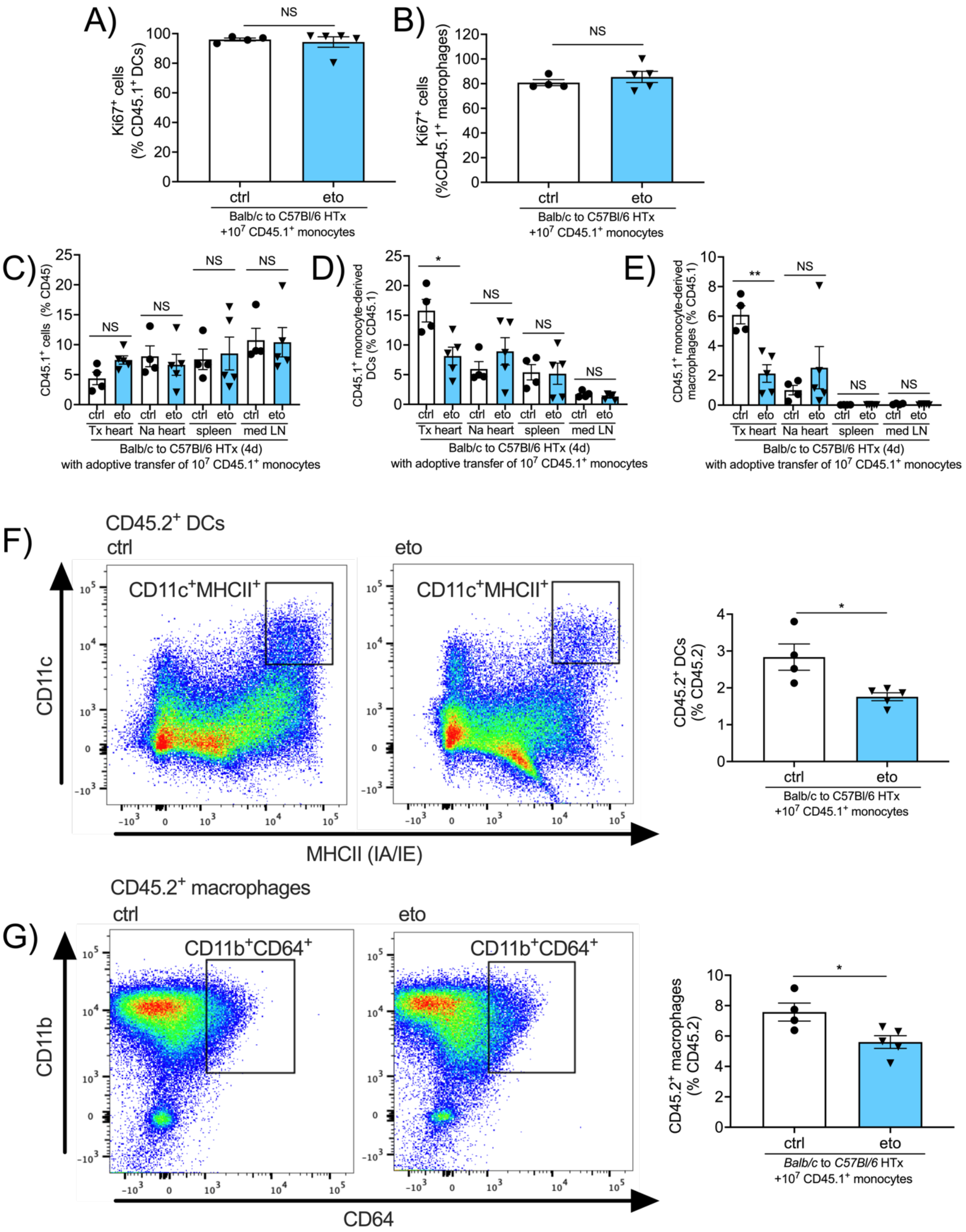
A)
NS
Ki67+ cells (% CD45.1+ DCs)
100
80
60
40
20
0
ctrl
eto
Balb/c to C57Bl/6 HTx
+10^7 CD45.1+ monocytes
B)
NS
Ki67+ cells (%CD45.1+ macrophages)
120
100
80
60
40
20
0
ctrl
eto
Balb/c to C57Bl/6 HTx
+10^7 CD45.1+ monocytes
C)
CD45.1+ cells (% CD45)
25
20
15
10
5
0
NS
NS
NS
NS
ctrl eto ctrl eto ctrl eto ctrl eto
Tx heart Na heart spleen med LN
Balb/c to C57Bl/6 HTx (4d)
with adoptive transfer of 10^7 CD45.1+ monocytes
D)
CD45.1+ monocyte-derived DCs (% CD45.1)
25
20
15
10
5
0
*
NS
NS
NS
ctrl eto ctrl eto ctrl eto ctrl eto
Tx heart Na heart spleen med LN
Balb/c to C57Bl/6 HTx (4d)
with adoptive transfer of 10^7 CD45.1+ monocytes
E)
CD45.1+ monocyte-derived macrophages (% CD45.1)
10
8
6
4
2
0
**
NS
NS
NS
ctrl eto ctrl eto ctrl eto ctrl eto
Tx heart Na heart spleen med LN
Balb/c to C57Bl/6 HTx (4d)
with adoptive transfer of 10^7 CD45.1+ monocytes
F)
CD45.2+ DCs
ctrl
eto
CD11c+MHCII+
CD11c+MHCII+
CD11c
MHCII (IA/IE)
CD45.2+ DCs (% CD45.2)
4
3
2
1
0
*
ctrl
eto
Balb/c to C57Bl/6 HTx
+10^7 CD45.1+ monocytes
CD45.2+ macrophages
G)
ctrl
eto
CD11b+CD64+
CD11b+CD64+
CD11b
CD64
CD45.2+ macrophages (% CD45.2)
10
8
6
4
2
0
*
ctrl
eto
Balb/c to C57Bl/6 HTx
+10^7 CD45.1+ monocytes

SUPPLEMENTAL FIGURE 4: ASSESSMENT OF CD45.1$^+$ MONOCYTES AND MONOCYTE, DC AND MACROPHAGE PROLIFERATION IN ADOPTIVE TRANSFER EXPERIMENTS. A-J) Flow cytometric assessment of transplanted hearts at 4 days post-HTx from Balb → B6 HTx adoptively transferred with CD45.1$^+$ monocytes. **A,B)** Assessment of proliferation by percentage Ki67 incorporation. **A)** In CD11c$^+$MHCII$^+$CD64$^-$ dendritic cells (DCs) as a percentage of CD45.1$^+$ cells (ctrl, n=4; eto, n=5). **B)** In CD11b$^+$CD64$^+$CD24$^-$ macrophages as a percentage of CD45.1$^+$ (ctrl, n=4; eto, n=5). **C)** CD45.1$^+$ cells as a percentage of CD45$^+$ cells in the transplanted heart (Tx heart), native heart (Na heart), spleen and mediastinal lymph node (med LN) (ctrl, n=4; eto, n=5). **D)** CD11c$^+$MHCII$^+$CD64$^-$ dendritic cells (DCs) as a percentage of CD45.1$^+$ cells in the Tx heart, Na heart, spleen and med LN (ctrl, n=4; eto, n=5). **E)** CD11b$^+$CD64$^+$CD24$^-$ macrophages as a percentage of CD45.1$^+$ cells in the Tx heart, Na heart, spleen and med LN (ctrl, n=4; eto, n=5). **F)** CD45.2$^+$ DCs assessed by CD11c$^+$MHCII$^+$CD64$^-$ cells (ctrl, n=4; eto, n=5). **G)** CD45.2$^+$ macrophages assessed by CD11b$^+$CD64$^+$CD24$^-$ cells (ctrl, n=4; eto, n=5). Data are represented as mean ± SEM. NS, not statistically significant; *p<0.05; NS, not statistically significant, by *t*-test (**A,B, F,G**) or ANOVA and Bonferroni post-hoc test (**C-E**).

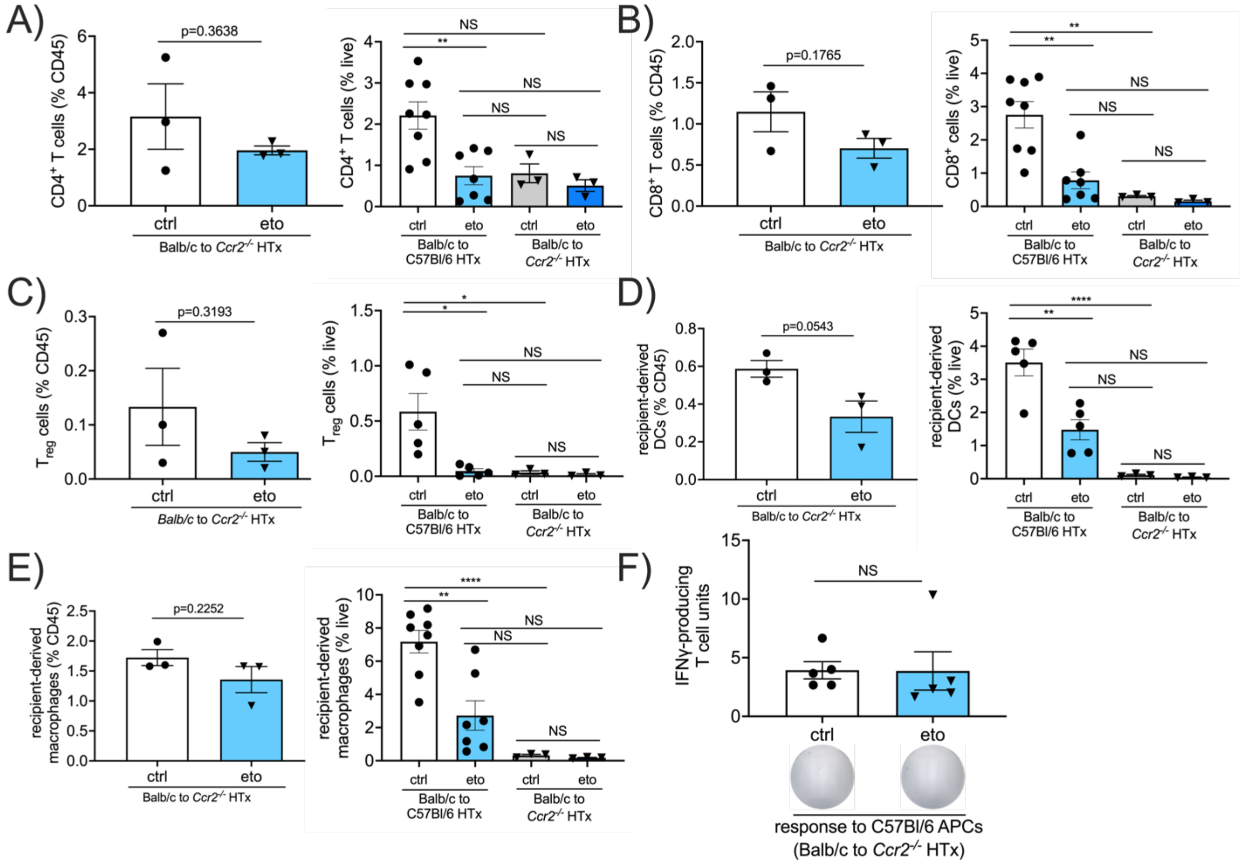

Supplemental Figure 5: Flow cytometry for T cells in *Ccr2*$^{-/-}$ HTx. A-E) Flow cytometric assessment in transplanted hearts from Balb → *Ccr2*$^{-/-}$ heart allografts at 4 days post-transplant shown as a percentage of CD45$^+$ cells (left graph) or live cells (right graph; data for live cells from Balb → B6 HTx is re-plotted from Figs. 1 and 2). **A)** CD4 T cells assessed by CD4$^+$CD3$^+$CD90.2$^+$ cells (ctrl, n=8; eto=7 for Balb → B6 HTx; ctrl, n=3; eto, n=3 for Balb → B6 *Ccr2*$^{-/-}$ HTx). **B)** CD8 T cells assessed by CD8$^+$CD3$^+$CD90.2$^+$ cells (ctrl, n=8; eto=7 for Balb → B6 HTx; ctrl, n=3; eto, n=3 for Balb → B6 *Ccr2*$^{-/-}$ HTx). **C)** T regulatory cells assessed by Foxp3$^+$CD4$^+$CD3$^+$CD90.2$^+$ cells (ctrl, n=8; eto=7 for Balb → B6 HTx; ctrl, n=3; eto, n=3 for Balb → B6 *Ccr2*$^{-/-}$ HTx). **D)** Recipient-derived dendritic cells assessed by IA-b$^+$CD11c$^+$MHCII$^+$CD64$^-$ cells (ctrl, n=5; eto=5 for Balb → B6 HTx; ctrl, n=3; eto, n=3 for Balb → B6 *Ccr2*$^{-/-}$ HTx). **E)** Recipient-derived macrophages assessed by IA-b$^+$CD11b$^+$CD64$^+$CD24$^-$ cells (ctrl, n=8; eto=7 for Balb → B6 HTx; ctrl, n=3; eto, n=3 for Balb → B6 *Ccr2*$^{-/-}$ HTx). **F)** ELISPOT assay performed using splenocytes procured at 4d post-transplantation from Balb → *Ccr2*$^{-/-}$ heart allografts from either vehicle (ctrl) or etomoxir (eto)-treated recipients. Samples were stimulated with activated B6 APCs and assessed for the production of IFNγ (ctrl, n=4; eto, n=4). Data are represented as mean ± SEM. NS, not statistically significant; $^{**}p<0.01$; $^{***}p<0.001$; $^{****}p<0.0001$ by *t*-test (**A-E left panels; F**) or ANOVA and Bonferroni post-hoc test (**A-E right panels**).

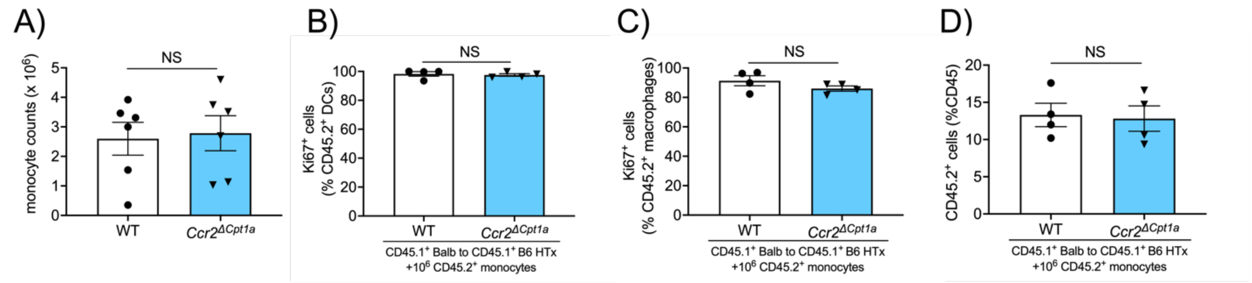

SUPPLEMENTAL FIGURE 6: GENETIC DELETION OF CPT1A ON PROLIFERATION OF MONOCYTE-DERIVED MACROPHAGES AND MONOCYTE-DERIVED DCS IN HEART ALLOGRAFTS. A) Cell counts after monocyte isolation from the bone marrow of *Cre-negative;Cpt1a*$^{fl/fl}$ (WT; n=6) or *Ccr2.Cre*^{ER}*;Cpt1a*$^{fl/fl}$ (*Ccr2*$^{\Delta Cpt1a}$; n=6) mice. **B-C)** Assessment of proliferation by percentage Ki67 incorporation in $CD45.2^+$ fraction from transplanted hearts in Balb to B6 $CD45.1^+$ HTx recipients adoptively transferred with WT or *Ccr2*$^{\Delta Cpt1a}$ monocytes. **B)** In $CD11c^+MHCII^+CD64^-$ dendritic cells (DCs) as a percentage of $CD45.2^+$ cells (WT, n=4; *Ccr2*$^{\Delta Cpt1a}$, n=4). **C)** In $CD11b^+CD64^+CD24^-$ macrophages as a percentage of $CD45.2^+$ (WT, n=4; *Ccr2*$^{\Delta Cpt1a}$, n=4). **D)** In $CD45.2^+$ cells as a percentage of $CD45^+$ cells (WT, n=4; *Ccr2*$^{\Delta Cpt1a}$, n=4). Data are represented as mean ± SEM. *p<0.05; NS, not statistically significant, by *t*-test.

SUPPLEMENTAL TABLE

antibody	clone	company
CD11b	M1/70	eBioscience (San Diego, CA)
CD3ε	17A2	eBioscience
CD4	GK1.5	eBioscience
CD45R (B220)	RA3-6B2	eBioscience
CD64	X54-5/7.1	eBioscience
CD90.2 (Thy-1.2)	30-12	eBioscience
Ki-67	SolA15	eBioscience
Ly-6G	1A8	eBioscience
MHC class II (I-A/I-E)	114.15.2	eBioscience
NK1.1	PK136	eBioscience
CD11b	M1/70	BD Biosciences (San Jose, CA)
CD11c	N418	BD Biosciences
CD11c	HL3	BD Biosciences
CD115	T38-320	BD Biosciences
CD24	M1/69	BD Biosciences
CD3ε	145-2C11	BD Biosciences
CD45	30-F11	BD Biosciences
CD45.1	A20	BD Biosciences
CD45.2	104	BD Biosciences
CD64	X54-5/7.1	BD Biosciences
CD69	H1.2F3	BD Biosciences
CD8a	53-6.7	BD Biosciences
Foxp3	M23	BD Biosciences
I-A[b]	AF-1201	BD Biosciences
Ly-6G	1A8	BD Biosciences
MHC class II (I-A/I-E)	M5/114.15.2	BD Biosciences
NK1.1	PK136	BD Biosciences
CD11c	N418	Biolegend (San Diego, CA)
Foxp3	FJK-16s	Thermo Scientific (Waltham, MA)

SUPPLEMENTAL TABLE 1. LIST OF ANTIBODIES USED FOR FLOW CYTOMETRY
